# Tree species identity, canopy structure and prey availability differentially affect canopy spider diversity and trophic composition

**DOI:** 10.1007/s00442-023-05447-1

**Published:** 2023-09-14

**Authors:** Benjamin Wildermuth, Clemens Dönges, Dragan Matevski, Alice Penanhoat, Carlo L. Seifert, Dominik Seidel, Stefan Scheu, Andreas Schuldt

**Affiliations:** 1 Department of Forest Nature Conservation, University of Göttingen, Büsgenweg 3, 37077 Göttingen, Germany; 2 Animal Ecology, Leuphana University Lüneburg, Universitätsallee 1, 21335 Lüneburg, Germany; 3 Department for Spatial Structures and Digitization of Forests, University of Göttingen, Büsgenweg 1, 37077 Göttingen, Germany; 4 Animal Ecology Group, JF Blumenbach Institute of Zoology and Anthropology, University of Göttingen, Untere Karspüle 2, 37073 Göttingen, Germany; 5 Centre of Biodiversity and Sustainable Land Use, University of Göttingen, Büsgenweg 1, 37077 Göttingen, Germany

**Keywords:** Araneae, Arboreal arthropods, Functional diversity, LiDAR, Mixed-species forestry, Trophic niche

## Abstract

Forest canopies maintain a high proportion of arthropod diversity. The drivers that structure these communities, however, are poorly understood. Therefore, integrative research connecting tree species identity and environmental stand properties with taxonomic and functional community composition of canopy arthropods is required. In this study, we investigated how the taxonomic, functional and trophic composition of arboreal spider communities is affected by tree species composition and associated differences in canopy structure and prey availability in temperate forests. We sampled canopy spiders as well as their potential prey using insecticidal fogging in monospecific and mixed stands of native European beech, native Norway spruce and non-native Douglas fir. Trophic metrics were obtained from stable isotope analysis and structural canopy properties were assessed with mobile laser scanning. Monospecific native spruce stands promoted local canopy spider abundance and diversity, but native beech and beech–conifer mixtures had the highest diversity at landscape scale. Spider community composition differed between monospecific stands, with broadleaf–conifer mixtures mitigating these differences. Irrespective of tree species identity, spider abundance, taxonomic diversity, functional richness and isotopic richness increased in structurally heterogeneous canopies with high prey abundances, but functional evenness and trophic divergence decreased. Our study shows that canopy spiders are differentially affected by tree species identity, canopy structure and prey availability. Broadleaf–conifer mixtures mitigated negative effects of (non-native) conifers, but positive mixture effects were only evident at the landscape scale. Structurally heterogeneous canopies promoted the dominance of only specific trait clusters. This indicates that intermediate heterogeneity might result in high stability of ecological communities.

**Supplementary Information:**

The online version contains supplementary material available at 10.1007/s00442-023-05447-1.

## Introduction

Forests are important safeguards of biodiversity in times of global biodiversity loss (Seibold et al. 2019; Hill et al. 2019). Yet, arthropods as the most diverse group of eukaryotes (Stork 2018) are on the decline in forest ecosystems (Seibold et al. 2019; Staab et al. 2023). Much of this arthropod diversity depends on forest canopies as habitats (Nakamura et al. 2017), but because tree canopies are hard to access, their associated biodiversity remains understudied, especially in temperate forests (Ulyshen 2011; Floren et al. 2022). Compared to the more intensively studied forest floor and subcanopy (see Burrascano et al. 2021), where stand characteristics such as canopy openness and herb cover play key roles in structuring arthropod communities (Ziesche and Roth 2008; Kriegel et al. 2021), canopy arthropods are commonly more directly affected by tree species identity (Pedley et al. 2014; Floren et al. 2022).

Diversification of tree communities can increase associated biodiversity of forests and improve forest adaptability to changing environments (Wagner et al. 2014; Ampoorter et al. 2020). Especially broadleaf–conifer mixtures have commonly been reported to show positive mixture effects due to their phylogenetic differences resulting in complementary functional effects (Schwarz and Bauhus 2019; Haberstroh and Werner 2022). In Central Europe, the naturally dominating broadleaved beech (*Fagus sylvatica* L.) and fast-growing Norway spruce (*Picea abies* (L.) H.Karst.) as an economically important tree, are promising candidates for such mixtures (Pretzsch et al. 2012). Further, non-native tree species are increasingly considered for climate change-adapted management (Thurm and Pretzsch 2016). Particularly, the Northern American Douglas fir (*Pseudotsuga menziesii* (Mirbel) Franco) is broadly considered as suitable for Central Europe (Thomas et al. 2022). However, non-native tree species could potentially threaten local biodiversity and ecosystem functioning (Tallamy et al. 2021), calling for research on ecological consequences (Schmid et al. 2014; Thomas et al. 2022).

Previously, broadleaf–conifer mixtures were often shown to mitigate rather than promote arthropod diversity (Barsoum et al. 2014; Oxbrough et al. 2016; Matevski and Schuldt 2023). Non-native trees were reported to have negative effects mostly on diversity and abundance of herbivorous arthropods (Tallamy et al. 2021; Berthelot et al. 2023). It is expected that non-native trees host no or few specialist arthropods in their new range (Roques et al. 2006), and as generalists are less efficient in their use of resources compared to specialists, the same resources may sustain less individuals (García et al. 2018). Yet, negative effects of non-native trees were less evident for predatory arthropods when only considering abundances and taxonomic diversity (Oxbrough et al. 2016; Matevski and Schuldt 2023), whereas expanding the scope to functional divergence and trophic complexity unraveled negative effects (Wildermuth et al. 2023; Matevski and Schuldt 2023). However, most studies to date have focused on lower forest strata, especially the forest floor, and the effects of non-native trees on canopy fauna are still little understood (Gossner and Ammer 2006).

While for herbivorous canopy arthropods, tree species identity and non-nativeness with their specific resources are major drivers for diversity and abundance (Leidinger et al. 2021; Tallamy et al. 2021), arboreal predators such as spiders are expected to rely more on canopy structure and general food availability (Korenko et al. 2011). Surprisingly, tree species identity often has stronger effects on arthropod predator community composition than local stand structure or prey availability (Mupepele et al. 2014). Nonetheless, structural heterogeneity of forest stands is known to generally increase abundance and diversity of associated arthropods (Müller et al. 2018; Rappa et al. 2023), e.g., due to increasing availability and diversity of habitats and resources via higher space filling (Müller et al. 2018). However, the effects of canopy structure have so far mostly been investigated for impacts on taxonomic arthropod community composition (Heidrich et al. 2020; Ramos et al. 2022). Better linking of tree identity and canopy properties with arthropods and their ecological role requires information on functional community composition and trophic interactions (Haddad et al. 2009; Cadotte et al. 2011).

Canopy structural heterogeneity is assumed to play a particularly important role for spiders (Araneae), as they rely on available structures for web attachment or shelter (Halaj et al. 2000; Korenko et al. 2011; Butz et al. 2023). Conifers in particular feature beneficial fine-scale structures and high prey abundances, increasing spider abundances compared to broadleaved trees (Ozanne 1999; Korenko et al. 2011). Arthropod predators such as spiders are important links in food webs, providing pest control by an estimated extent greater than that provided by birds, while being an important food source for birds themselves (Nyffeler et al. 2018). In Europe, 25–30% of the spider fauna is associated with forests (Blick et al. 2019). Yet, canopy-associated spiders are sparsely studied, although up to 40% of forest spider species live predominantly in the canopy (Otto and Floren 2007). The key ecological role of spiders therefore calls for increased consideration of canopy spiders and their functioning for our basic ecological understanding and applications such as nature conservation and forest management (Pedley et al. 2016; Milano et al. 2021). In their functioning as top-down control agents, spider trophic niches are a particularly important proxy for trophic interactions, with broader feeding niches and more complex trophic interactions possibly increasing the resilience of the whole system (Poisot et al. 2013; Michalko and Pekár 2016). Stable isotope analysis is a method that has become increasingly popular to study trophic interactions (Potapov et al. 2019). Natural isotope ratios of ^13^C/^12^C (δ^13^C) and ^15^N/^14^N (δ^15^N) reflect trophic positions and dietary sources (Post 2002). In animal tissue, δ^13^C and δ^15^N differ depending on the food sources: ^13^C concentrations differ between basal carbon sources (e.g., leaves, detritus or microbes), and ^15^N is enriched at each trophic level (Post 2002). Therefore, dual analysis of δ^13^C and δ^15^N allows to determine animal trophic niches (Potapov et al. 2019). Previous research has shown that the trophic niches of ground-dwelling spiders can be altered and simplified by admixture of broadleaved forests with non-native conifers, while conifers generally promote spider isotopic richness (Wildermuth et al. 2023).

In this study, we investigated how the taxonomic, functional and trophic composition of arboreal spider communities is affected by changes in tree species composition and associated differences in canopy structure and prey availability in temperate forests. A comprehensive sampling of canopy spiders requires elaborate and rarely deployed sampling techniques which enable capturing non-flying as well as flying taxa (Floren 2010; Floren et al. 2022). We therefore used insecticidal fogging to study mixed and monospecific stands of native European beech, native Norway spruce and non-native Douglas fir. The trophic niches of spiders were subsequently analyzed using stable isotope analyses. We assessed structural canopy features with high-resolution mobile laser scanning. We hypothesized that (i) native Norway spruce, but not non-native Douglas fir, promotes spider abundance, biomass, functional richness and isotopic richness compared to European beech. We further hypothesized that (ii) canopy spiders show tree species identity-dependent differences in community composition and trophic niche, and that (iii) broadleaf–conifer mixtures mitigate tree species identity effects. Lastly, we hypothesized that (iv) independent of tree species identity, canopy structural heterogeneity has strong positive effects on spider taxonomic and functional richness.

## Methods

### Study site

The 20 study plots were located in the managed, temperate Solling forest in Lower Saxony, Germany (N51.666, E9.569; 300 m.a.s.l.; Appendix S1: Fig. S1, Table S1). The climate of the study area is characterized by mean annual temperatures of 7–8 °C and mean annual precipitation between 800 and 950 mm (NIBIS® Kartenserver 2021). The forest is dominated by naturally regenerating European beech (*F. sylvatica*) and planted Norway spruce (*P. abies*), with a small proportions of monospecific and admixed stands of planted non-native Douglas fir (*P. menziesii*). We investigated five stand types: monocultures of European beech, Douglas fir and Norway spruce and the mixtures of European beech with each of the two conifers. We sampled four plots of each stand type resulting in a total of 20 plots. Sampling plots consisted of four to six trees which were fogged. The mean distance between plots was 1066 m (± 619 m; standard error), with a minimum distance of 100 m. Plot locations were chosen outside of protected areas and with consideration of plot accessibility with the fogging machine and low exposure to wind. Across plots, average tree age was 50.3 ± 6.3 years (Appendix S1, Table S1). Plots had equal proportions of trees of the admixed species and low densities of understory vegetation. The canopies in each plot slightly overlapped. Stem densities and tree heights varied due to limited suitable sampling locations. These structural differences, however, were accounted for and analyzed statistically. Due to the small proportion of planted Douglas fir and suitable sampling locations across the forest, Douglas fir plots were located in small patches, surrounded by beech or Norway spruce. The highly targeted fogging sampling, however, ensured exclusive sampling of Douglas fir in these plots.

### Arthropod data

#### Arthropod and leaf sampling

We fogged all plots between May 31 and July 3, 2021 under dry and windless conditions, using the thermal fog generator Swingfog SN 50 (Swingtec, Isny, Deutschland) and 1% natural pyrethrum solution. We chose this sampling time because the active periods of most Central European spider species cover June (Nentwig et al. 2021). The targeted canopy area was effectively fogged for 5–10 min. We placed four white collecting sheets of 2 × 3 m as closely grouped as possible to each plot to collect falling arthropods from the canopy. We raised the collecting sheets on poles to ~ 1 m above ground to prevent ground-associated arthropods from entering (Floren 2010). After each fogging, we waited for 2 h of drop-down time. Using fine brushes, we carefully swept all arthropods per collecting sheet together and stored them in 70% ethanol. We excluded one Douglas fir plot (1.2) from all subsequent analyses due to inadequate fogging. We sampled canopy leaves for the trophic baseline calibration on all plots, using a slingshot and a manual rope chain saw. We cut off one branch from the mid-canopy (~ 15–20 m) in one tree of each tree species per plot.

#### Identification, functional diversity and prey availability

We identified all adult spiders to species level and derived the mean body lengths of females and males, using established keys and online sources (Appendix S1). Based on these size measurements, we estimated biomasses for all male and female specimens individually, using the linear regression from Penell et al. (2018). Specimens which could not be identified to species level were excluded from our analyses. We assigned all species to their guilds (orb, sheet and space web weavers; ambush, and other hunters) after Cardoso et al. (2011) and derived the phenological length (in months) of active adult spiders from Nentwig et al. (2021). Guild and phenology of activity are key determinants of spider resource use, and therefore of their functional impact (Cardoso et al. 2011).

Using the R package “FD” (Laliberté et al 2014), we calculated the following functional diversity indices based on guild, phenology and mean biomass (Cardoso et al. 2011) at the plot level: unweighted functional richness (FRic) and relative biomass-weighted functional divergence (FDiv) and functional evenness (FEve; Villéger et al. 2008, see Appendix S1 for further explanation). As a proxy for prey availability, we counted Diptera, Hymenoptera and Sternorrhyncha in each sample, which together make up > 90% of spider prey in temperate forests (Nentwig 1985).

#### Stable isotope analysis

We analyzed the trophic niche structure of spider species comprising the top 80% of abundance per plot, reflecting the dominant community and thus the main functional impact (Krause et al. 2021). Isotopic analysis of carbon and nitrogen ratios (δ^13^C and δ^15^N) was conducted by the Centre for Stable Isotope Research Analysis at the University of Göttingen. For detailed description of spider and leaf sample (baseline) preparation and the stable isotope analysis pipeline see Appendix S1. To compensate for variation in the isotopic baseline between plots, we calibrated all spider isotopic ratios with mean *δ* values of leaves from the respective plot (Lu et al. 2022). Calibrated ratios henceforth are denoted as Δ^13^C and Δ^15^N.

We calculated all isotopic metrics of spider communities at the plot level. As one-dimensional isotopic metrics, we included isotopic mean, minimum, maximum and range of Δ^13^C and Δ^15^N. Minimum and maximum values can reveal extreme values, indicating unique basal resources, and the isotopic ranges reflect on the breadth of used resources and trophic levels, i.e., the diversity of consumed prey (Krause et al. 2021). Equivalent to the functional diversity indices, multidimensional isotopic metrics included unweighted isotopic richness (IRic) and relative biomass-weighted isotopic divergence (IDiv) and isotopic evenness (IEve). Further, we analyzed the isotopic uniqueness (IUni). These indices are based on Villéger et al. (2008) and were adapted for isotopic values by Cucherousset and Villéger (2015). See Appendix S1 for further explanation of the multidimensional isotopic metrics.

#### Forest structural data

Within 1 week after arthropod sampling, we scanned each plot with a mobile laser scanner (Zeb-Horizon, Geoslam, Nottingham, UK). Acquiring 300,000 points per second, the scanner has an accuracy of up to 3 cm and a maximum range of 100 m. Holding the scanner in the hand, we moved in a spiral trajectory from the center of the fogging area to the outside, ensuring a buffer of 2 m around the fogged area.

The calculated indices of tree structure were the overall vegetation volume, the mean effective number of vertical canopy layers (ENL), mean horizontal canopy gap area (mean gap area), the coefficient of variation of horizontal canopy gap area per plot (CV gap area), the CV of intra-canopy gap height in the canopy per plot (CV ICG height) and the box dimension as a measure of structural complexity (Seidel 2018). For the quantification of intra-canopy gaps, we adapted the quantification of horizontal gaps for the empty space within the canopy. Structural canopy properties such as vertical layering, gap sizes and three-dimensional complexity are important drivers of forest arthropods, including spiders (Halaj et al. 2000; Heidrich et al. 2020). We initially calculated all structural variables for cropped point clouds of radii from 1 to 12 m from the sampling center, using the R package “LidR” (Roussel et al. 2020). We observed that the stand structural complexity reached an asymptote at 9 m radius on all fogging sites. Therefore, we decided to only consider a radius of 9 m around the center of the fogging area in our analyses. This radius included the sampling area plus an approximate 4 m buffer. For detailed descriptions of the point cloud processing, see Appendix S1.

### Statistical analyses

All subsequent statistical analyses were conducted with R 4.2.1 (R Core Team 2022). We used the package “vegan” (Oksanen et al. 2022) to calculate spider species richness, effective number of species and evenness on sample and plot level. We split our analyses into two steps, (i) analyzing stand type effects on spider responses and environmental properties (prey abundance and structural attributes) while integrating total vegetation volume in the model and (ii) analyzing the effects of canopy structural properties and prey availability, while integrating stand type as random effect. We integrated vegetation volume into the stand type analysis to correct for biases by differing sampled tree volumes, as canopy fogging is a highly targeted sampling approach with defined spatial sampling extent (Floren 2010). Including stand type as random effect in (ii) allowed to analyze environmental variables across stand types.

In the modeling step (i), we analyzed all spider responses (abundance, biomass, diversity indices, functional diversity indices, isotopic metrics) and structural stand attributes at the plot level in linear models with stand type and vegetation volume as fixed effects (vegetation volume was not included in the analysis of structural stand attributes). Further, we extended this model for analyses at the sample level (per collecting sheet) to a linear mixed-effects model, with spider abundance, biomass, diversity and prey abundance as response and including plot as random effect, using the R package “lme4” (Bates et al. 2014). We applied pairwise Tukey HSD post hoc tests for stand type comparisons, using the “multcomp” package (Hothorn et al. 2008). In modeling step (ii), we analyzed spider responses at plot level in linear mixed-effects models with structural properties and prey abundance as fixed effects and stand type as random effect. As in (i), we added a sample-level analysis for abundance, biomass and diversity and included plot and stand type as crossed random effects. For further details on model selection and fitting, see Appendix S1.

To investigate the influence of stand type on spider richness at the landscape scale, we used abundance-based rarefaction and short-range extrapolation of the species richness at stand level across plots, and at the level of pooled mixed and monoculture stands across stand types. This approach is based on effective species numbers and allows for robust abundance-weighted diversity estimates for double the sample size (Hsieh et al. 2016). We conducted this analysis for the Hill numbers *q* = 0, 1 and 2, using the “iNEXT” package (Hsieh et al. 2016). Hill number *q* = 0 is equivalent to species richness, *q* = 1 reflects the effective number of species (the exponent of the Shannon diversity) and *q *= 2 reflects the number of dominant species (inverse Simpson diversity; Hsieh et al. 2016).

To investigate differences in spider community composition at sample and plot scale between stand types, we applied two-dimensional nonmetric multidimensional scaling (NMDS), using the package “vegan” (Oksanen et al. 2022). We used the Morisita–Horn index for distance estimations, which emphasizes dominant species and thus is resistant to undersampling (Magurran and McGill 2011). Using the NMDS axes scores (“envfit” function), structural properties were fitted post hoc to the ordination. Implementing an analysis of similarity (ANOSIM, *N* = 9999) with post hoc pairwise comparison, using the package “pairwiseAdonis” (Martinez Arbizu 2017), we tested for significant differences in community composition between stand types. To investigate whether individual stand types are characterized by particular spider species, we conducted indicator species analyses, using the package “labdsv” (Roberts 2019) and considered only species with at least ten recorded individuals.

## Results

We sampled 815 undamaged adult spider specimens comprising 45 species (Appendix S1, Table S2). They represented five guilds, with most spiders being web weavers: space web (Dictynidae, Theridiidae) and orb web weavers (Araneidae, Tetragnathidae) comprised 79.8% of all individuals (650/815) and 48.9% of all species (22/45). These proportions were similar across stand types (Appendix S1: Table S2). Prey abundances were significantly higher in monospecific spruce (1170 ± 104), beech–spruce mixtures (490 ± 90) and monospecific Douglas fir (1014 ± 341) than in monospecific beech stands (74 ± 6; *F*_(4,13.9)_ = 3.14, *p* < 0.001; *p* < 0.01; *p* < 0.01). Further, prey abundances were higher in monospecific spruce than in beech–Douglas fir mixtures (330 ± 77; *p* < 0.05). Structural stand attributes at plot level did not differ significantly between stand types, but in trend structural complexity and vertical layering were highest in spruce, and mean gap area was highest in Douglas fir (Appendix S1: Table S3).

### Abundance, taxonomic diversity and community composition

**Fig. 1:**
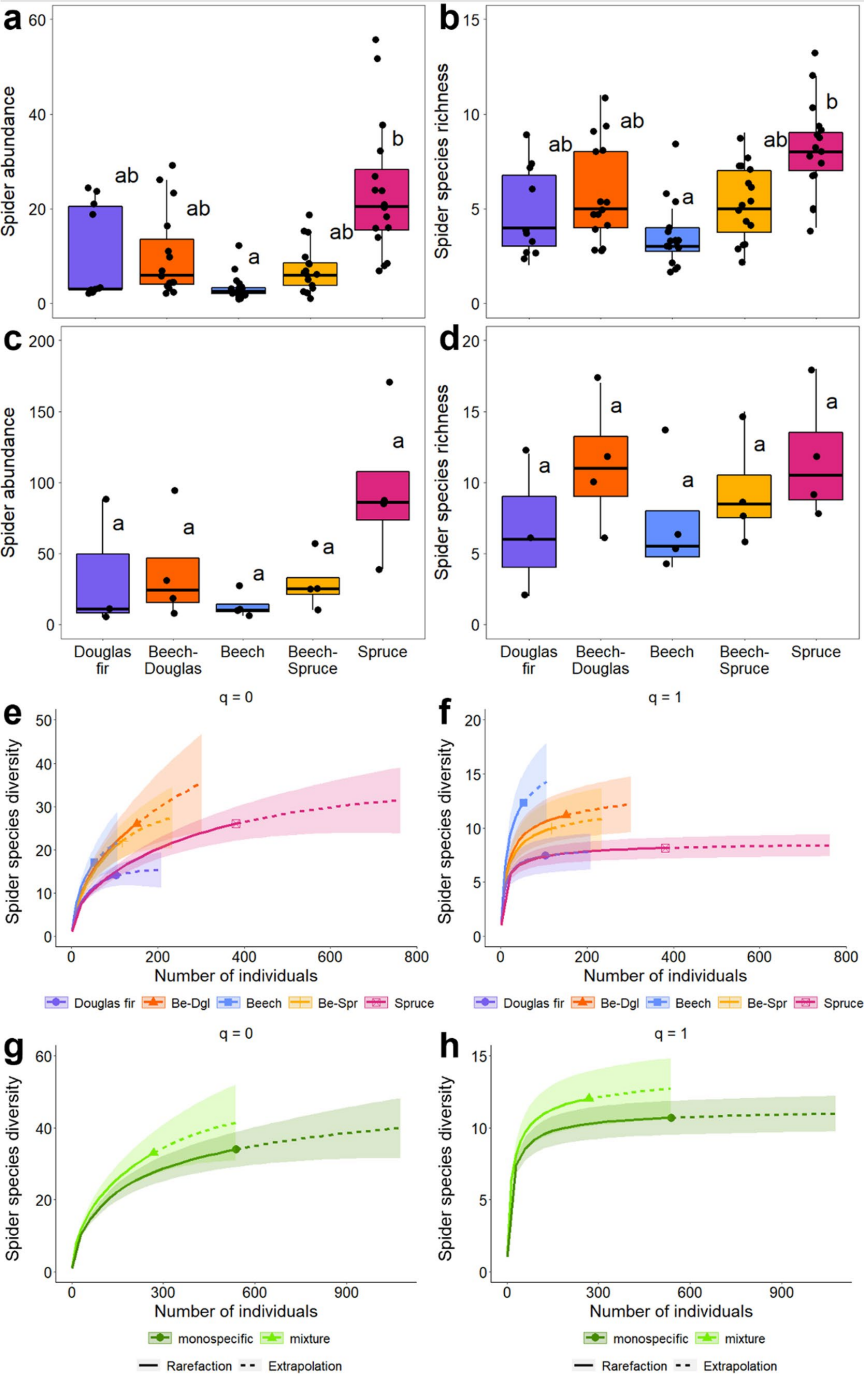
**a**, **b** Local (sample-level) spider abundance and richness per stand type, **c**, **d** plot-level spider abundance and richness per stand type (not significant), **e**, **f** abundance-based species accumulation and extrapolation curves at stand scale with *q* = 0, 1, **g**, **h** abundance-based accumulation and extrapolation curves at landscape scale with *q* = 0, 1. Shaded areas represent 95% confidence bands

At the sample level (per collecting sheet = local), monospecific spruce stands harbored significantly higher spider abundances (Fig. 1a) and biomass (48.1 mg ± 12.7) than monospecific beech (*F*_(4,12.9)_ = 3.53, *p* < 0.005; 3.15 mg ± 0.92, *F*_(4,12.8)_ = 3.88, *p* < 0.005). Further, local diversity was significantly higher in monospecific spruce than in monospecific beech (species richness: *F*_(4,12.9)_ = 2.87, *p* < 0.05; Fig. 1b; effective number of species: *F*_(4,12.8)_ = 2.36, *p* < 0.05; Appendix S1: Table S4). At plot level, spider abundances also were higher in monospecific spruce than in monospecific beech stands, but this difference was only marginally significant (*F*_(4)_ = 2.88, *p* = 0.08; Fig. 1c). Spider biomass and diversity did not differ significantly between stand types at the plot level (Fig. 1d, Appendix S1: Table S5).

Species accumulation and extrapolation curves at the landscape scale based on Hill numbers showed that monospecific and mixed beech stands had the highest species diversity, whereas monospecific conifer stands had significantly lower species numbers (Fig. 1e, f). Overall, mixtures tended to have higher spider species numbers than monocultures (non-significant; Fig. 1g, h).

**Fig. 2:**
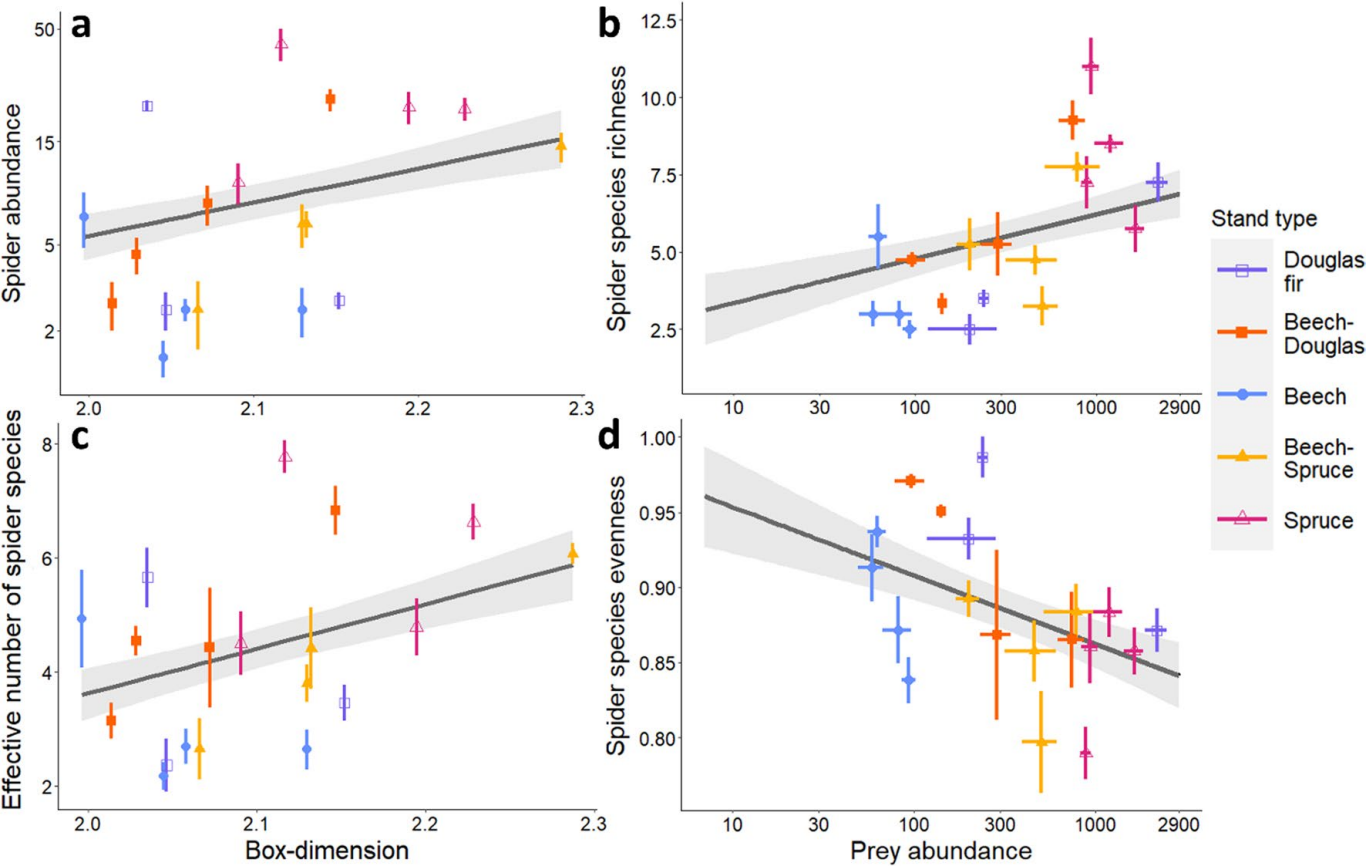
Relationships between box dimension (structural complexity) and local (sample-level) **a** spider abundance and **c** effective number of species ± standard error. Relationships between prey abundance (log-transformed) and local **b** species richness, and **d** evenness

Vegetation volume neither correlated significantly with local and plot-level canopy spider abundance nor diversity. Spider abundance, biomass and species richness correlated positively with structural complexity (box dimension; Fig. 2a), effective number of vegetation layers (ENL), the variability of intra-canopy gap heights (CV ICG height) and prey abundance (Fig. 2b). Box dimension, ENL and CV ICG height further correlated positively with the spider effective number of species (Fig. 2c). Spider species evenness decreased with increasing variation of horizontal canopy gap area and prey availability (Fig. 2d), but increased with *mean gap area* (Appendix S1: Table S6). At plot level, the patterns were largely the same (Appendix S1: Table S7).

**Fig. 3:**
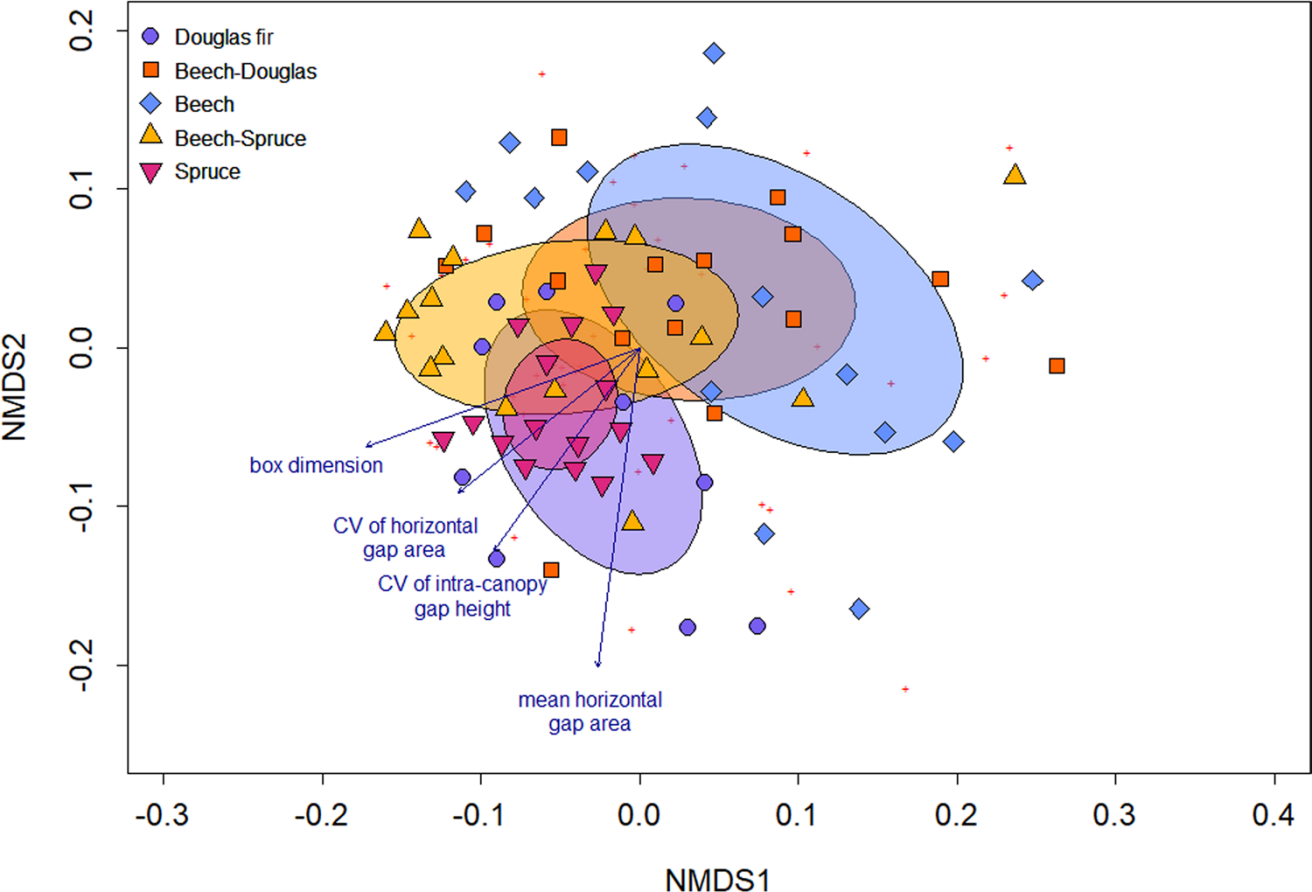
Nonmetric multidimensional scaling (NMDS) ordination of spider community composition per stand type. Stress < 0.2. Red crosses represent spider species. Filled symbols represent study plots and stand types. Ellipses show standard deviation of stand type point scores. Blue arrows show significant correlations of environmental variables with axes scores

Spider communities at the local scale differed significantly between all monospecific stands, with spruce monocultures also differing from both mixture types (ANOSIM *p* < 0.05 for all comparisons). While beech–Douglas fir mixtures did not differ significantly from monospecific beech and Douglas fir stands, or from spruce–beech mixtures, the latter differed significantly from monospecific beech and spruce stands (*p* < 0.05; Appendix S1; Table S8). Box dimension, CV gap area and CV ICG height correlated with spider communities that were characteristic for coniferous stands. Mean gap area correlated with typical Douglas fir communities (Fig. 3, Appendix S1: Table S9). At the plot scale, stress values of the NMDS rose to > 0.25 and no significant differences between stand types could be observed.

The indicator species analysis revealed no significant indicator species in Douglas fir and beech–conifer mixtures. Monospecific beech stands had one marginally significant indicator species (*Nigma flavescens* (Walckenaer, 1830); *p* = 0.1) which is a space web weaver. Spruce stands had three significant indicator species (*Anelosimus vittatus* (C. L. Koch, 1836), *Philodromus collinus* C. L. Koch, 1835, *Platnickina tincta* (Walckenaer, 1802); for all *p* < 0.05), representing two space web weavers and one hunting spider (*P. collinus*).

### Functional diversity

**Fig. 4:**
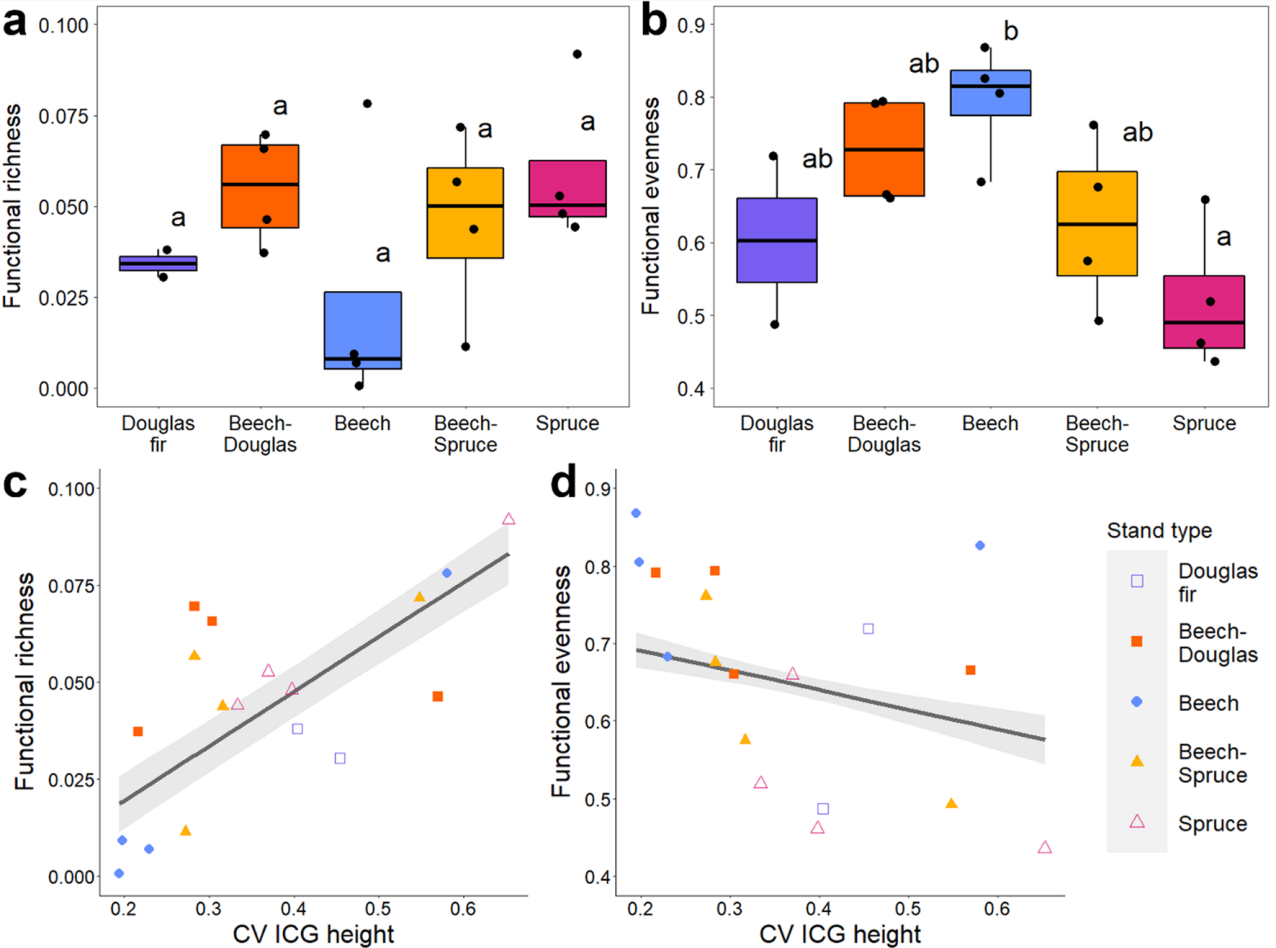
**a**, **b** Functional richness and evenness per stand type and **c**, **d** their relationships with CV ICG height

Most functional indices showed non-significant trends between stand types (Fig. 4a, Appendix S1: Table S10). Only spider functional evenness differed significantly between stand types, with higher evenness in monospecific beech than in monospecific spruce stands (*F*_(4)_ = 4.26, *p* < 0.05; Fig. 4b).

Spider functional richness (FRic) correlated positively with total vegetation volume, ENL, mean gap area and CV ICG height (Fig. 4c). Further, FRic correlated negatively with CV gap area. Functional evenness (FEve) correlated negatively with CV ICG height (Fig. 4d) and prey abundance, and positively with CV gap area (Appendix S1: Tables S10, S11).

### Trophic niches

**Fig. 5:**
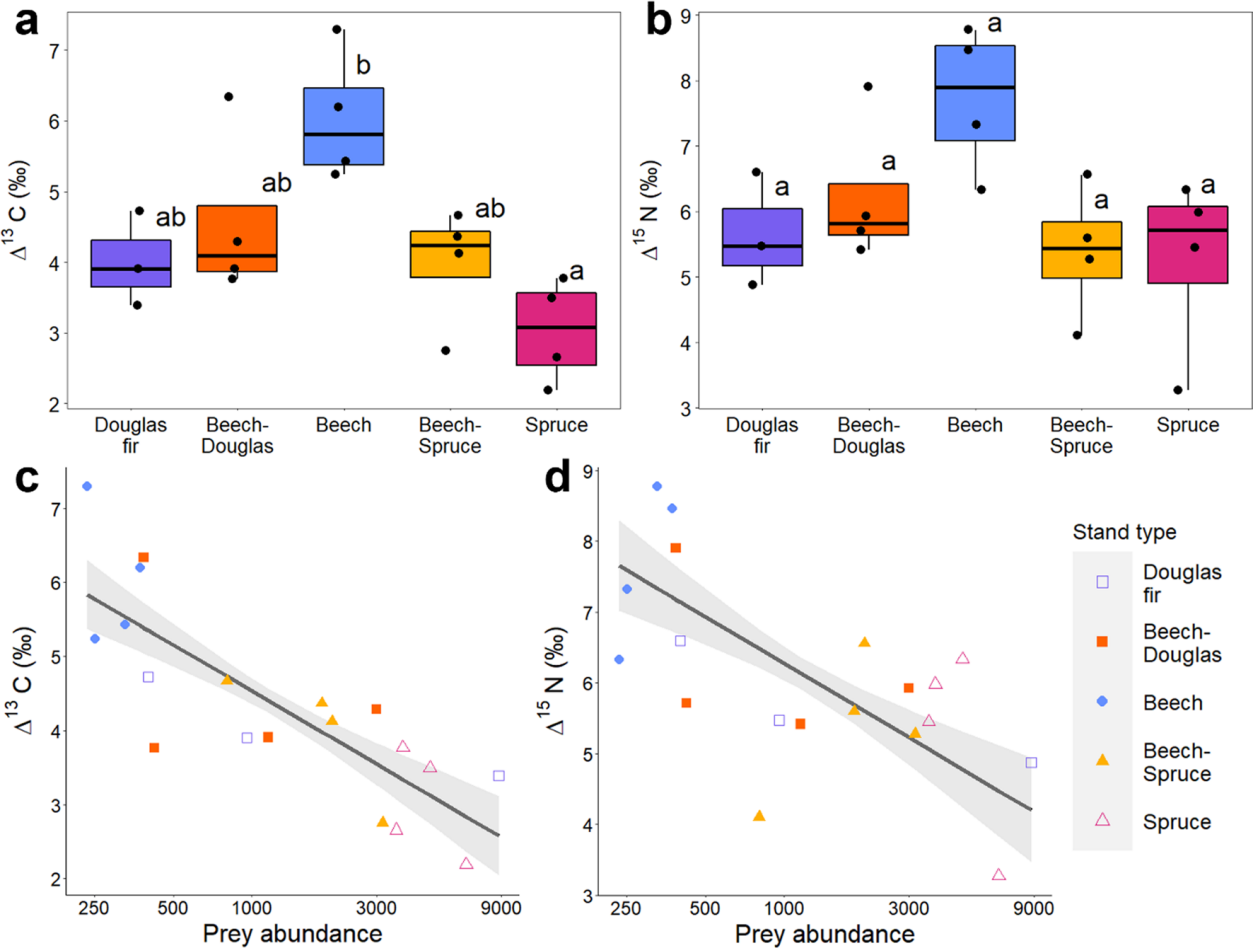
Mean ∆^13^C and ∆^15^N per stand type (**a**, **b**) and their relationships with prey abundance (**c**, **d**; log-transformed)

Mean ∆^13^C of spiders was significantly higher by 3.01 ‰ in monospecific beech stands compared to monospecific spruce stands (*p* < 0.01; Fig. 5a), and in trend higher by 2.03 ‰ compared to monospecific Douglas fir stands (*F*_(4)_ = 5.5, *p* = 0.12; not significant). Further, minimum and maximum ∆^13^C of spiders were significantly higher in beech than in spruce stands, with minimum ∆^13^C also being higher in beech compared to beech–spruce mixtures. In trend, mean spider ∆^15^N was higher by 2.46 ‰ in monospecific beech stands compared to monospecific spruce stands (*F*_(4)_ = 2.95, *p* = 0.11; not significant; Fig. 5b). Multidimensional isotopic metrics of spider trophic niches did not differ significantly between stand types (Appendix S1: Table S12).

**Fig. 6:**
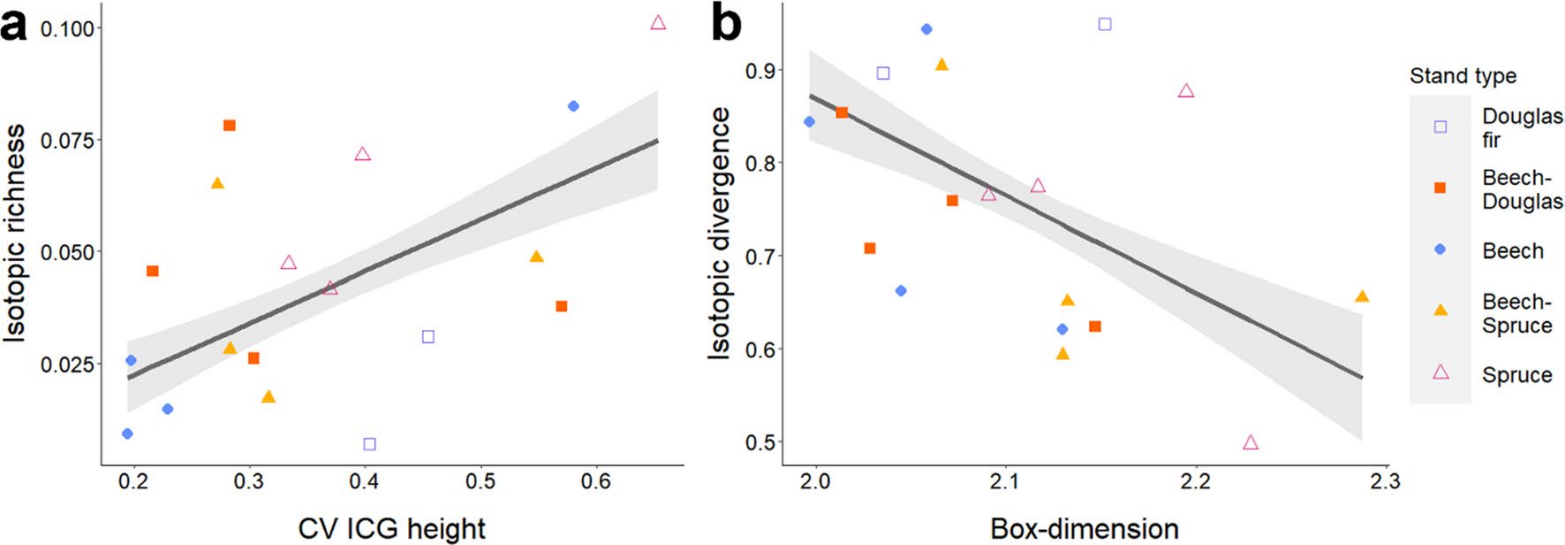
Relationships between **a** CV ICG height and isotopic richness (IRic) and **b** box dimension (structural complexity) and isotopic divergence (IDiv)

No isotopic metric correlated significantly with total vegetation volume. Mean, minimum and maximum ∆^13^C and ∆^15^N of spiders correlated negatively with prey abundance (Fig. 5c, d). The ranges of spider ∆^13^C and ∆^15^N correlated negatively with CV gap area. Further, range ∆^13^C of spiders correlated positively with mean gap area and CV ICG height, and isotopic richness (IRic) with CV ICG height (Fig. 6a). In contrast, isotopic divergence (IDiv) of spiders correlated negatively with box dimension (Fig. 6b), but positively with mean gap area (Appendix S1: Table S13).

## Discussion

Our results suggest that at local scales, native Norway spruce, but not non-native Douglas fir, promotes canopy spider abundance and diversity. At landscape scale, however, native beech and beech–conifer mixtures showed the highest species turnover and therefore highest overall species richness. Beech–conifer mixtures further mitigated differences in spider community composition between beech and conifer monocultures. These results indicate that broadleaf–conifer mixtures may maintain canopy spider diversity and native communities in European beech forests. Monospecific patches of conifers, however, host distinct spider communities and are only beneficial for spider abundance and diversity locally and only in case of native conifers. Independent of tree species identity, structurally heterogeneous canopies increased spider abundance, diversity, and functional richness, but very high levels of heterogeneity led to dominance of few spider traits (low functional evenness and isotopic divergence). This indicates that intermediate rather than very high heterogeneity might stabilize canopy spider communities via balanced richness and evenness of traits.

### Stand type effects on taxonomic, functional and trophic diversity

At the local scale, canopy abundance and diversity were promoted by native spruce compared to native broadleaved beech, but not by non-native Douglas fir (Fig. 1a, b). This supports our first hypothesis that native conifers promote spider abundance and diversity but, opposed to findings on the forest floor (Matevski and Schuldt 2023), non-native conifers do not. We suggest that in the canopy, tree species identity effects are more important (Gossner and Ammer 2006; Floren et al. 2022), while beneficial effects of conifers on generalist predators on the forest floor are strongly linked to general stand characteristics like light availability and herb cover (Ziesche and Roth 2008; Kriegel et al. 2021). However, also in the canopy general positive effects of conifers on spider communities were observed (Ozanne 1999; Korenko et al. 2011).

The question to ask is therefore which tree species identity effects are leading to the lack of positive conifer effects in the canopy of non-native Douglas fir. However, our study only found non-significant trends of higher stand-scale structural complexity and prey availability, and smaller canopy gaps in spruce compared to non-native Douglas fir. Therefore, either unmeasured structural factors may be key drivers of the observed differences in spider communities, or our number of sampling plots was too small to detect stand-scale differences in structure. Possible unmeasured drivers could be differences in microscale structure, such as bark texture or needle density (Halaj et al. 1998; Blick and Gossner 2006), which might reduce suitability or number of structures for web building and shelter (Oxbrough et al. 2005; Korenko et al. 2011). In combination with (non-significant) macroscale structural differences, such as less dense canopies, this may impact overwintering of spiders and their prey organisms, with strongly reduced arthropod abundances in non-native Douglas fir in winter compared to native spruce (Gossner and Utschick 2004). These seasonality effects might also drive the observed low abundances in broadleaved beech, where many arthropods are forced to move to the ground after leaf drop, which will also reduce canopy arthropod numbers in summer (Gil 2009).

Another possible driver of the lack of positive conifer effects in Douglas fir might be the lack of shared evolutionary history with native prey and spider communities (Tallamy et al. 2021). Douglas fir was shown to only host a fraction of its specialized associated arthropods when planted in Europe (Roques et al. 2006), and herbivorous arthropods are generally profoundly reduced and altered in their community composition by non-native plants compared to native plants (Tallamy et al. 2021; Berthelot et al. 2023). While we acknowledge that generalist predators should not be strongly impacted by tree species-specific community composition of their prey, we emphasize that tree-specific spider communities in our and previous studies cannot simply be explained by structure and prey availability (Mupepele et al. 2014). Further, spiders indeed can show prey specialization (García et al. 2018; Mezőfi et al. 2020). In line with this, we did not record any indicator species on Douglas fir, while native spruce hosted three significant indicator species. Yet, these indicator species should theoretically benefit from both conifer species, as *Philodromus collinus* is generally associated with conifers and the space web weaving indicator species (*Anelosimus vittatus*, *Platnickina tincta*) should benefit from the needles of either species to attach their small-scale webs (Halaj et al. 1998; Mupepele et al. 2014). We conclude that tree species identity effects beyond structure and general prey availability may determine that non-native conifers do not support the same local diversity of canopy spiders as native conifers (Pedley et al. 2014). Notably, prey abundances were several times higher in spruce than in native beech, which may indicate that high spider abundances do not necessarily imply improved pest control, but rather reflect high abundances of potential pest species.

Supporting our second hypothesis, tree species identity effects in our study were further underlined by pronounced differences in spider community composition between monospecific stands (Fig. 3). This concurs with previous studies, showing that conifers are associated with different arthropod communities than native broadleaved forests (Pedley et al. 2014; Matevski and Schuldt 2020; Kriegel et al. 2021). In line with this, the indicator species of monospecific beech stands, *Nigma flavescens*, is a specialized space web weaver, building its nets on leaves of broadleaved trees (Nentwig et al. 2021). Again, this emphasizes that also for generalist species, tree species identity can be a strong driver of community composition and, supported by our indicator species analysis, tree species-specific spiders do exist in the canopy (Mupepele et al. 2014).

In line with our third hypothesis, local differences in species richness and abundance between spruce and beech were mitigated in mixtures of the two tree species (Fig. 1a, b). Further, spider community compositions in mixed stands were intermediate between the respective monospecific stands, mitigating tree species identity effects (Fig. 3). This supports previous studies on the forest floor, reporting mitigating effects of tree species mixtures on generalist predator abundance and diversity (Kriegel et al. 2021; Matevski and Schuldt 2023), indicating averaging trade-off effects (van der Plas et al. 2016). However, while beneficial effects of spruce on spider abundance and diversity became smaller at the plot scale, they reversed at the landscape scale: both conifers markedly showed the lowest diversity, whereas mixed and monospecific beech stands had the highest spider diversity (Fig. 1e, f). When pooling all monospecific and all mixed stands, we even observed a general positive mixture effect at the landscape scale (Fig. 1g, h). This corroborates reports of low spatial arthropod species turnover in conifers (Oxbrough et al. 2016; Matevski and Schuldt 2020) and indicates beneficial effects of tree diversification (Ampoorter et al. 2020; Matevski and Schuldt 2020). Notably, we recorded nine species exclusively in mixed stands, but their low abundances do not allow robust conclusions on their uniqueness to mixtures. Comparing our results on canopy spiders to previous studies on the forest floor suggests that tree species identity and their admixture differentially impact forest floor- and canopy-associated arthropods (Pedley et al. 2016). In conclusion, conservation of canopy arthropod communities in European forests can only be safeguarded when native broadleaves are admixed.

Partially contradicting our first hypothesis, we did not observe significant differences in functional or isotopic richness between stand types (Fig. 4a), suggesting that major canopy spider functional groups can be sustained by all investigated stand types and that the spider communities feed on similar ranges of prey (Michalko et al. 2019). However, spider functional evenness was higher in beech than in spruce stands, indicating that the high abundances in spruce result in only few trait clusters, whereas the few spiders in beech have very different functions (Fig. 4b). This supports findings on the forest floor, showing that monospecific conifer plantations lead to functional homogenization of spiders (Matevski and Schuldt 2023). Further, we found marked differences in the trophic niches of canopy spiders between stand types, with ∆^13^C and ∆^15^N being the highest in monospecific beech stands and low in coniferous stands (Fig. 5a, b). Notably, the ∆^13^C pattern is the same as observed on the forest floor, while ∆^15^N contrasts patterns of ground-dwelling spiders (Wildermuth et al. 2023). High ∆^13^C values indicate that the food web is fueled by detrital resources (“detrital shift”; Potapov et al. 2019), whereas low ∆^13^C values indicate a rather herbivore-fueled food web (Krause et al. 2021; Wildermuth et al. 2023). This suggests that prey organisms in conifer canopies predominantly consume decaying material, microbes and fungi, while prey in beech stands mostly consume living leaves (Pollierer et al. 2023). However, the observed ∆^13^C patterns may also reflect different feeding strategies of tree species specific herbivore communities, such as the selective use of easy to digest plant compounds with high ∆^13^C (Pollierer et al. 2023). High ∆^15^N values in beech stands hint toward an additional trophic level compared to coniferous stands (Scheu and Falca 2000), likely reflecting more pronounced intra-guild predation in beech (Wildermuth et al. 2023). In fact, prey abundances were lowest in beech stands and had a negative relationship with ∆^15^N (Fig. 5d). Such a top-heavy food web as in beech may be explained by the phenology of deciduous trees, which do not provide resources for herbivores during winter (Pollierer et al. 2023). This also indicates that pest control provided by spiders may be more pronounced in beech than in coniferous stands, even though spiders are more abundant in conifers. Tree species mixtures mitigated these effects, supporting our second hypothesis and consolidating their potential as buffer against potentially negative tree species identity effects.

### Habitat heterogeneity and prey availability

Despite not differing significantly between stand types, we identified forest stand structure as a strong driver of spider communities, supporting our fourth hypothesis. Heterogeneous heights of intra-canopy gaps, high vertical layering and structural complexity promoted spider abundance and diversity, coupled with positive effects of prey abundance (Fig. 2a–c). Previous studies suggested that especially non-flying taxa such as spiders benefit from structurally complex environments (Halaj et al. 2000; Ramos et al. 2022). Some studies even reported that canopy structure explains more variance in canopy spider abundance and species richness than prey availability (Halaj et al. 1998; Butz et al. 2023). Structural complexity and vertical vegetation layering promote arthropod abundances and diversity via higher space-filling, which increases total habitat and resource availability (Müller et al. 2018; Knuff et al. 2020; Rappa et al. 2023). However, although canopy gaps are known to be crucial for forest arthropod diversity, previous studies mostly investigated gaps interrupting the canopy (Heidrich et al. 2020; Junggebauer et al. 2021). Yet, the canopy is a three-dimensional habitat, and intra-canopy gaps should be considered, as for instance also removal of low canopy structures will alter the available habitat (Dial et al. 2006). The variation in intra-canopy gap height had a strong positive relationship with multiple spider responses, emphasizing the advantages of assessing canopy structure with high-resolution laser scanning (Müller et al. 2018; Seidel 2018). We propose that variation in intra-canopy gap height promotes vertical availability of hunting grounds, while—in combination with vertical layering—spatial separation of these habitats is assured (Müller et al. 2018; Knuff et al. 2020).

Structurally heterogeneous canopies further promoted spider functional and isotopic richness (Figs. 4c, 6a), suggesting that habitat heterogeneity increases the total trait space via increasing habitat availability and habitat diversity (Stein and Kreft 2015). However, heterogeneous canopies also decreased functional evenness and isotopic divergence, indicating promotion of trait clusters in the functional and trophic niche space respectively (Figs. 4d, 6b). This effect was also coupled with increasing prey availability (Fig. 2d). Further, variability of horizontal gap sizes decreased spider functional richness and isotopic ranges, indicating a limited trophic diversity of consumed prey. This may indicate that high structural complexity promotes functional similarity of spiders via spatial partitioning and increased abundance of prey with high trophic similarity (Müller et al. 2018; Tsang et al. 2023). Yet, this similarity can be mediated by canopy gaps, as we also found an increase in contrasting trophic traits (isotopic divergence) with larger horizontal gap size, and increasing functional evenness with variation in horizontal gap size. This suggests that, although structurally heterogeneous canopies might not increase all functional spider traits evenly, with resource availability promoting dominant traits, canopy gaps and their heterogeneity in size can foster functional dissimilarity. The latter underlines that multiple heterogeneity attributes might have differing relationships with canopy arthropods and that in sum, intermediate habitat heterogeneity might balance positive and negative impacts (Heidrich et al. 2020; Swart et al. 2020). This would also lead to higher ecological stability of communities via even occupation of dissimilar niches (Godoy et al. 2018; Tsang et al. 2023).

## Conclusions

Our study shows that canopy spider communities are differentially driven by tree species identity, canopy structure and prey availability. As spider community composition strongly differed depending on tree species identity and species turnover was low in non-native trees, native trees should be of great conservational interest. However, broadleaf–conifer mixtures showed buffering against negative tree species identity effects, while promoting spider diversity at the landscape scale. Monospecific plantations of non-native Douglas fir in Central Europe should therefore not be recommended, but admixtures with native beech potentially promote canopy spider diversity, indicating that they may be a more suitable management option when also considering biodiversity conservation. Structurally heterogeneous canopies and prey availability promoted the richness of spider species and traits, but it also fostered dominance of only few trait clusters. This indicates that heterogeneity-diversity relationships are not just linear positive, and intermediate heterogeneity might result in more stable ecological communities. Our study shows that findings of beneficial conifer effects from the ground stratum cannot be transferred to the canopy—even for generalist predators such as spiders—as tree species identity and intra-canopy structure are intimately linked with canopy spider communities.

### Supplementary Information

Below is the link to the electronic supplementary material.

Supplementary file1 (PDF 946 KB)

## Data Availability

Not applicable.

## Data availability

Data (Wildermuth 2023) are available from GRO.data: 10.25625/UMJIQA.
