# Supplementary material for: Tree species identity, canopy structure and prey availability differentially affect canopy spider diversity and trophic composition
Source: Oecologia. 2023 Sep 14;203(1-2):37–51. doi: 10.1007/s00442-023-05447-1 (PMC10615988; doi:10.1007/s00442-023-05447-1)
Supplement: Supplementary file 1 — Supplementary file1 (PDF 946 KB) [file 442_2023_5447_MOESM1_ESM.pdf]

Oecologia

Tree species identity, canopy structure and prey availability differentially affect canopy spider diversity and trophic composition

*Benjamin Wildermuth; Clemens Dönges; Dragan Matevski; Alice Penanhoat; Carlo L. Seifert; Dominik Seidel; Stefan Scheu; Andreas Schuldt

*Corresponding author: Benjamin Wildermuth; bmwildermuth6@gmail.com

Appendix S1

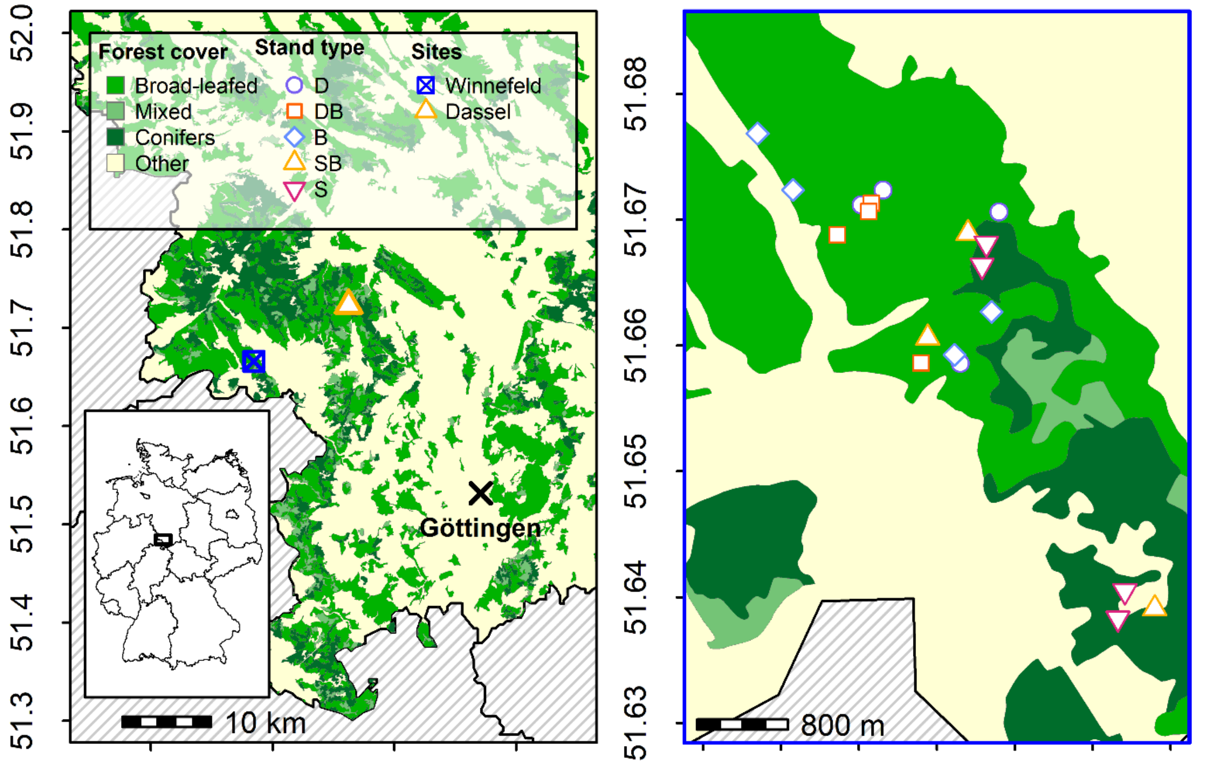

Appendix S1: Fig. S 1 Map of the study plots in the Solling forest. All plots except one spruce-beech mixture (4.3, see table S1) were located in the "Winnefeld" forest district (zoom in right panel), 4.3 was located in the "Dassel" forest district (see the overview in the left panel). D = Douglas fir, DB = Douglas fir-beech, B = beech, SB = spruce-beech, S = spruce.

Spider identification, functional indices

We identified all adult spiders to species-level, using the following established keys and online sources: Roberts (1993, 1996), Nentwig et al. (2021), Oger (2021), Wiki der Arachnologischen Gesellschaft e. V. (2021). Taxonomy and nomenclature follow the World Spider Catalogue (2022). We derived mean body lengths of females and males for each species from the Wiki der Arachnologischen Gesellschaft e. V. (2021).

The estimation of functional richness (FRic) is based on the total functional space occupied. Values between 0 and 1 represent the space filled by the community. Functional divergence (FDiv) measures the distance between species within the functional space. Values close to 0 indicate that the most abundant species have traits that are centered in the functional trait space, and values close to 1 represent dominance of traits which are distinct to each other. Functional evenness (FEve) quantifies the species value distribution in the functional space. If the values are evenly distributed, FEve is close to 1, whilst clustered values push FEve towards 0 (e.g. if most species have the same functional traits).

Stable isotope analysis

If multiple species with the same abundance added up to the top 80%, we selected the species with highest mean body mass according to Nentwig et al. (2021). Prior to stable isotope analysis, we discarded the opisthosoma and dried the spiders and tree leaves at 60° C for 72 h. The removal of the opisthosoma ensures the exclusion of isotopic bias due to recently digested material (Perkins et al. 2013). In case of large species, we bisected the prosoma longitudinally, whilst for very small species, we pooled multiple prosomas to reach the required minimum dry weight of ~ 0.05 mg. For leaf isotopic analyses, we pooled the tips and bases of two leaves per sampled tree.
An elemental analyzer (CE-Instruments, Rodano, Milano, Italy) coupled with an isotope ratio mass spectrometer (Delta XP, Thermo Electron, Bremen, Germany) was used. For small samples < 0.1 mg, the setup was adapted (Langel and Dyckmans 2014). Acetanilide (C_8H_9NO, Merck, Darmstadt) was used for internal calibration. Standards were Vienna PeeDee belemnite (C) and atmospheric nitrogen (N). Isotopic C and N values were given as deviation δ relative to the respective standards (Fry 2006), with $\delta^{15}N$ and $\delta^{13}C$ being defined as δX (‰) = $(R_{sample} - R_{standard})/R_{standard} \times 1000$. R equals the ratio between heavy and light isotope ($^{13}C/^{12}C$ or $^{15}N/^{14}N$).
All calculations of isotopic metrics are based on the convex hull area, covered by the isotopic ratios of the community (Layman et al. 2007). The interpretation is equivalent to the above described interpretation of functional indices. Further, isotopic uniqueness (IUni) reflects whether isotopic values within the community are unique or if they overlap. IUni approaches 1 when most isotopic values are unique, and IUni values close to 0 show high redundancy of isotopic values (e.g., if one trophic position is covered by multiple species).

Point cloud processing

Based on the laser scanning point cloud, we calculated the overall *vegetation volume*, the mean effective number of vertical canopy layers (*ENL*), mean horizontal canopy gap area (*mean gap area*), the coefficient of variation of horizontal canopy gap area per plot (*CV gap area*), the CV of intra-canopy gap height in the canopy per plot (*CV ICG height*) and the *box-dimension* as a measure of structural complexity. To calculate these variables, we cropped the point cloud to radii from 1-12 m, using the R package "LidR" (Roussel et al. 2020). We subsampled each point cloud using the space tool in CloudCompare, to remove points closer than 0.5 cm to each other. Thereafter, we normalized the point cloud with a digital terrain model as implemented in "LidR". After voxelizing the point clouds to voxels of 50 cm side length, we classified voxels as empty if they contained no point, and non-empty if they contained at least one point, with each point being a laser hit.

We considered non-empty voxels as vegetation and calculated the *vegetation volume* as the sum of all vegetation voxels times their volume. We calculated the *ENL0* following the method of Ehbrecht et al. (2016). The *ENL0* represents the number of non-empty voxels in the vertical voxel column. We calculated the *ENL0* for each voxel column in the cropped point cloud and averaged it at plot level. For the calculation of horizontal gaps-interrupting the canopy-we considered contiguous cells with no vegetation above two meters as gaps (Brokaw 1982). Using the R package "ForestGapR" (Silva et al. 2019) we calculated the *mean* and *CV gap area* per plot. As canopies do not only feature horizontal gaps interrupting the canopy, but also gaps within the canopy, we adapted the quantification of horizontal gaps for the empty space within the canopy. For the calculation of these "intra-canopy gaps" (ICGs), we connected all neighboring empty voxels within the canopy (in horizontal and vertical directions) which had no connection to the empty space above or below the canopy to form 3D gaps. We calculated the *CV ICG height* per plot. As a measure of structural complexity, we calculated the box-dimension (Seidel 2018). To calculate this fractal index, we fitted the canopy point cloud in boxes of successively decreasing size, starting with a box the size of the bounding box of the point-cloud containing all points, up to a box of 10 cm size length (lower cut-off). For each box size, the number of boxes necessary to englobe all points are counted. The box dimension corresponds to the slope of the regression of the logarithm of the number of boxes as a function of the logarithm of the inverse of the box size. Further algorithmic details can be found in Arseniou et al. (2021).

Model selection and fitting

Using the variance inflation factor (VIF), we assured that no multicollinearity between fixed effects was given (VIFs < 5; Fox and Weisberg 2019). In the modelling step (ii), we reduced the models to the essential predictors based on the *step* function in the "lmerTest" R package (Kuznetsova et al. 2017). Because prey abundance had a strong positive skewedness, we log-transformed it to avoid disproportionally high impact of high values (Fink 2009). Due to non-normal residual distribution we log-transformed spider abundance and biomass values for analyses at the sample-level.

Appendix S1: Table S 1 Stand age classes and coordinates of the study plots. In case of mixtures, age class 1 refers to the coniferous species and age class 2 refers to beech. Age class 3 is always referring to admixed age classes of beech.

Plot	Stand type	Age class 1	Age class 2	Age class 3	longitude	latitude
1.1	Douglas	45			9.563005	51.658586
1.2	Douglas	59			9.567963	51.670631
1.3	Douglas	42			9.55031	51.671179
1.4	Douglas	29			9.553088	51.672357
2.1	Beech-Douglas	42	61		9.551586	51.671319
2.2	Beech-Douglas	42	61	45	9.551265	51.670641
2.3	Beech-Douglas	29	178	23	9.54723	51.668798
2.4	Beech-Douglas	35	35		9.557955	51.658621
3.1	Beech	45			9.562261	51.659261
3.2	Beech	45			9.5670169	51.6626884
3.3	Beech	181	34		9.5415519	51.6723663
3.4	Beech	59			9.537004	51.676853
4.1	Beech-Spruce	13	32		9.587953	51.639098
4.2	Beech-Spruce	46	44		9.558835	51.660609
4.3	Beech-Spruce	50	43		9.724863	51.723077
4.4	Beech-Spruce	46	50		9.563993	51.66892
5.1	Spruce	54	29		9.583238	51.638317
5.2	Spruce	29			9.584121	51.640496
5.3	Spruce	37			9.566349	51.668114
5.4	Spruce	46			9.565797	51.66635

Appendix S1: Table S 2 Guilds and abundances of all identified and analysed spider species per stand type. Web weavers are split into *orb* web weavers, *sheet* web weavers and *space* web weavers. Hunting spiders are split into *ambush* hunters and other *hunters*.

Family	Species	Guild	Douglas	Beech-Douglas	Beech	Beech-Spruce	Spruce
Anyphaenidae	*Anyphaena accentuata*	*Hunter*	1	1	2	1	2
Araneidae	*Araneus sturmi*	*Orb*	3	1	0	0	1
	Araniella alpica	*Orb*	0	1	0	0	0
	Araniella cucurbitina	*Orb*	1	3	6	3	1
	Araniella displicata	*Orb*	0	1	0	1	0
	Araniella opisthographa	*Orb*	0	1	1	0	0
	Cyclosa conica	*Orb*	0	3	1	0	2
	Gibbaranea omoeda	*Orb*	0	0	0	0	2
Clubionidae	*Clubiona brevipes*	*Hunter*	0	2	0	0	0

	Clubiona comta	*Hunter*	0	1	0	1	0
Dictynidae	*Dictyna pusilla*	*Space*	0	0	0	1	1
	Lathys humilis	*Space*	2	0	0	0	7
	Nigma flavescens	*Space*	2	7	10	4	0
Linyphiidae	*Agyneta conigera*	*Sheet*	1	0	0	0	0
	Agyneta innotabilis	*Sheet*	0	1	0	0	1
	Diplocephalus cristatus	*Hunter*	0	1	0	0	0
	Diplocephalus picinus	*Hunter*	0	1	0	0	0
	Dismodicus elevatus	*Hunter*	0	0	0	0	1
	Entelecara congenera	*Hunter*	0	0	0	1	7
	Erigonella hiemalis	*Hunter*	0	0	0	0	1
	Gongylidiellum latebricola	*Hunter*	0	0	0	1	0
	Moebelia penicillata	*Hunter*	1	0	0	0	0
	Neriene emphana	*Sheet*	0	0	2	0	0
	Neriene peltata	*Sheet*	4	2	7	2	2
	Nusoncus nasutus	*Hunter*	2	4	0	0	1
	Obscuriphantes obscurus	*Sheet*	0	0	0	2	0
	Tenuiphantes tenuis	*Sheet*	0	1	0	0	1
Philodromidae	*Philodromus albidus*	*Hunter*	2	1	0	3	0
	Philodromus aureolus	*Hunter*	0	0	0	0	2
	Philodromus collinus	*Hunter*	3	3	0	8	65
Salticidae	*Ballus chalybeius*	*Hunter*	0	2	3	2	0
Tetragnathidae	*Metellina mengei*	*Orb*	3	0	1	0	3
	Tetragnatha obtusa	*Orb*	27	32	2	16	78
Theridiidae	*Anelosimus vittatus*	*Space*	8	12	0	6	56
	Paidiscura pallens	*Space*	22	42	7	21	22
	Parasteatoda lunata	*Space*	0	0	1	1	3
	Parasteatoda simulans	*Space*	0	0	1	0	0
	Platnickina tincta	*Space*	30	15	4	37	106
	Robertus scoticus	*Space*	0	0	1	0	0
	Theridion boesenbergi	*Space*	0	0	0	1	8
	Theridion mystaceum	*Space*	0	6	0	2	4
	Theridion pinastri	*Space*	0	0	0	2	3
	Theridion varians	*Space*	0	0	1	0	0
Thomisidae	*Diaea dorsata*	*Ambush*	1	6	3	1	1
	Xysticus lanio	*Ambush*	0	1	0	0	0

Appendix S1: Table S 3 Mean values of all analyzed structural properties per stand type (± SE). CV = coefficient of variation; ICG = intra-canopy gaps.

Stand structure	Unit	Douglas	Beech-Douglas	Beech	Beech-Spruce	Spruce
Vegetation volume	10^6 m3	6.83 ± 1.06	5.84 ± 2.29	6.65 ± 8.46	6.21 ± 4.35	6.67 ± 3.36
Mean gap area	m2	2.59 ± 1.83	1.07 ± 0.51	0.13 ± 0.07	0.65 ± 0.3	0.82 ± 0.19
ENL0		21.79 ± 2.53	20.47 ± 1.17	21.32 ± 0.52	21.6 ± 1.44	25.3 ± 0.66
CV gap area		0.91 ± 0.51	0.87 ± 0.5	0 ± 0	0.76 ± 0.49	1.05 ± 0.15
CV ICG height		0.36 ± 0.07	0.34 ± 0.08	0.3 ± 0.09	0.35 ± 0.06	0.44 ± 0.07
Box-dimension		2.08 ± 0.04	2.07 ± 0.03	2.06 ± 0.03	2.15 ± 0.05	2.16 ± 0.03

Appendix S1: Table S 4 Model summaries of linear mixed-effects models of spider responses at the local scale (per collecting sheet) with stand type and vegetation volume as fixed effects.

Response	Fixed effect	Estimate	Std. Error	DF	t-value	P
log(Abundance)	(Intercept)	1.557	0.464	13.277	3.356	0.005
log(Abundance)	stand Be-Dgl	0.478	0.629	13.101	0.76	0.461
log(Abundance)	stand Beech	-0.671	0.604	13.082	-1.11	0.287
log(Abundance)	stand Be-Spr	0.223	0.613	13.063	0.364	0.722
log(Abundance)	stand Spruce	1.365	0.604	13.083	2.26	0.042
log(Abundance)	scale(vegetation volume)	0.311	0.195	12.803	1.59	0.136
log(Biomass)	(Intercept)	1.397	0.608	13.268	2.296	0.039
log(Biomass)	stand Be-Dgl	0.68	0.824	13.051	0.825	0.424
log(Biomass)	stand Beech	-0.793	0.791	13.027	-1.002	0.335
log(Biomass)	stand Be-Spr	0.308	0.803	13.003	0.383	0.708
log(Biomass)	stand Spruce	1.976	0.791	13.028	2.499	0.027
log(Biomass)	scale(vegetation volume)	0.434	0.255	12.682	1.701	0.113
Species richness	(Intercept)	4.224	1.219	13.432	3.466	0.004
Species richness	stand Be-Dgl	1.783	1.649	13.181	1.081	0.299
Species richness	stand Beech	-0.871	1.584	13.153	-0.55	0.592
Species richness	stand Be-Spr	1.142	1.607	13.125	0.711	0.49
Species richness	stand Spruce	3.741	1.583	13.154	2.363	0.034
Species richness	scale(vegetation volume)	0.664	0.511	12.752	1.3	0.216
Effective nr of species	(Intercept)	3.683	0.837	13.481	4.401	0.001
Effective nr of species	stand Be-Dgl	1.343	1.131	13.169	1.188	0.256
Effective nr of species	stand Beech	-0.694	1.086	13.131	-0.639	0.534
Effective nr of species	stand Be-Spr	0.641	1.101	13.097	0.582	0.57
Effective nr of species	stand Spruce	2.114	1.086	13.133	1.947	0.073
Effective nr of species	scale(vegetation volume)	0.527	0.349	12.631	1.508	0.156
Evenness	(Intercept)	0.929	0.03	14.252	31.095	<0.001
Evenness	stand Be-Dgl	-0.014	0.04	13.782	-0.339	0.74
Evenness	stand Beech	-0.039	0.039	13.713	-1.021	0.325
Evenness	stand Be-Spr	-0.07	0.039	13.659	-1.797	0.095
Evenness	stand Spruce	-0.082	0.039	13.715	-2.119	0.053
Evenness	scale(vegetation volume)	0.004	0.012	12.942	0.296	0.772

Appendix S1: Table S 5 Model summaries of linear models of spider responses at the plot-scale with stand type and vegetation volume as covariates.

Response	Fixed effect	Estimate	Std. Error	t-value	P
Abundance	(Intercept)	30.979	21.486	1.442	0.173
Abundance	stand Be-Dgl	11.99	29.245	0.41	0.688
Abundance	stand Beech	-19.787	28.104	-0.704	0.494
Abundance	stand Be-Spr	0.126	28.527	0.004	0.997
Abundance	stand Spruce	62.022	28.095	2.208	0.046
Abundance	scale(vegetation volume)	9.952	9.194	1.082	0.299
Biomass	(Intercept)	6.121	2.551	2.4	0.032
Biomass	stand Be-Dgl	5.9	3.472	1.699	0.113
Biomass	stand Beech	0.824	3.336	0.247	0.809
Biomass	stand Be-Spr	3.653	3.386	1.079	0.3
Biomass	stand Spruce	5.296	3.335	1.588	0.136
Biomass	scale(vegetation volume)	1.471	1.091	1.348	0.201

Species richness	(Intercept)	38.335	50.21	0.763	0.459
Species richness	stand Be-Dgl	23.347	68.342	0.342	0.738
Species richness	stand Beech	-30.259	65.675	-0.461	0.653
Species richness	stand Be-Spr	-2.299	66.664	-0.034	0.973
Species richness	stand Spruce	149.143	65.655	2.272	0.041
Species richness	scale(vegetation volume)	21.872	21.484	1.018	0.327
Effective nr of species	(Intercept)	4.194	1.274	3.291	0.006
Effective nr of species	stand Be-Dgl	3.171	1.734	1.829	0.091
Effective nr of species	stand Beech	1.502	1.667	0.901	0.384
Effective nr of species	stand Be-Spr	2.595	1.692	1.534	0.149
Effective nr of species	stand Spruce	1.78	1.666	1.068	0.305
Effective nr of species	scale(vegetation volume)	0.943	0.545	1.729	0.107
Evenness	(Intercept)	0.813	0.056	14.519	<0.001
Evenness	stand Be-Dgl	0.01	0.076	0.127	0.901
Evenness	stand Beech	0.078	0.073	1.061	0.308
Evenness	stand Be-Spr	0.035	0.074	0.469	0.647
Evenness	stand Spruce	-0.064	0.073	-0.874	0.398
Evenness	scale(vegetation volume)	-0.009	0.024	-0.367	0.72

Appendix S1: Table S 6 Model summaries of linear mixed-effects models of spider responses at the local scale (per collecting sheet) with environmental variables (prey abundance, mean gap area, ENL0, CV gap area, CV ICG height, Box-dimension) as fixed effects and stand type and plot as random effects. Model reduction (step function) might have reduced the number of fixed effects depending on their predictive power.

Response	Fixed effect	Estimate	Std. Error	DF	t-value	P
log(Abundance)	(Intercept)	1.88	0.174	2.631	10.797	0.003
log(Abundance)	scale(log(prey))	0.396	0.111	36.454	3.586	0.001
log(Abundance)	scale(box-dimension)	0.27	0.107	11.988	2.51	0.027
log(Abundance)	scale(ENL0)	0.452	0.11	13.529	4.114	0.001
log(Abundance)	scale(CV ICG height)	0.454	0.093	9.322	4.88	0.001
log(Biomass)	(Intercept)	1.891	0.226	2.721	8.351	0.005
log(Biomass)	scale(log(prey))	0.585	0.152	32.937	3.84	0.001
log(Biomass)	scale(box-dimension)	0.299	0.143	12.309	2.084	0.059
log(Biomass)	scale(ENL0)	0.59	0.147	13.821	4.028	0.001
log(Biomass)	scale(CV ICG height)	0.608	0.124	9.725	4.882	0.001
Species richness	(Intercept)	5.508	0.496	2.717	11.101	0.002
Species richness	scale(log(prey))	0.733	0.318	27.401	2.308	0.029
Species richness	scale(box-dimension)	0.81	0.283	10.845	2.86	0.016
Species richness	scale(ENL0)	1.175	0.292	12.467	4.028	0.002
Species richness	scale(CV ICG height)	1.233	0.243	8.754	5.074	0.001
Effective nr of species	(Intercept)	4.457	0.342	3.209	13.049	<0.001
Effective nr of species	scale(box-dimension)	0.589	0.207	12.888	2.845	0.014
Effective nr of species	scale(ENL0)	0.863	0.214	13.98	4.043	0.002
Effective nr of species	scale(CV ICG height)	0.985	0.183	10.639	5.391	<0.001
Evenness	(Intercept)	0.885	0.013	3.018	66.478	<0.001
Evenness	scale(log(prey))	-0.023	0.009	23.759	-2.484	0.02
Evenness	scale(mean gap area)	0.038	0.01	14.892	3.838	0.002
Evenness	scale(CV gap area)	-0.046	0.01	13.332	-4.592	<0.001
Evenness	scale(CV ICG height)	0.013	0.006	11.023	2.147	0.055

Appendix S1: Table S 7 Model summaries of linear mixed-effects models of spider responses at the plot-scale with environmental variables (prey abundance, mean gap area, ENL0, CV gap area, CV ICG height, Box-dimension) as fixed effects and stand type random effect. Model reduction (step function) reduced the number of fixed effects depending on their predictive power.

Response	Fixed effect	Estimate	Std. Error	DF	t-value	P
Abundance	(Intercept)	42.411	5.726	3.108	7.406	0.005
Abundance	scale(log(prey))	17.631	6.801	11.861	2.592	0.024
Abundance	scale(ENL0)	17.314	6.259	14.673	2.766	0.015
Abundance	scale(CV ICG height)	19.272	6.397	12.894	3.013	0.01
Bodymass	(Intercept)	9.402	0.758	3.44	12.411	0.001
Bodymass	scale(CV ICG height)	3.299	0.716	15.249	4.607	<0.001
Species richness	(Intercept)	67.794	14.989	16	4.523	<0.001
Species richness	scale(ENL0)	56.238	15.408	16	3.65	0.002
Species richness	scale(CV ICG height)	59.763	15.408	16	3.879	0.001
Effective nr of species	(Intercept)	6.098	0.42	17	14.532	<0.001
Effective nr of species	scale(CV ICG height)	1.29	0.431	17	2.991	0.008
Evenness	(Intercept)	0.825	0.024	4.222	33.877	<0.001

Appendix S1: Table S 8 Pairwise comparison of the species compositions between stand types (pairwiseAdonis).

Pairs (stand)	Degrees of freedom	Sums of squares	F-value	R2	P	Adjusted P
Douglas vs Be-Dgl	1	0.62	1.83	0.07	0.082	0.82
Douglas vs Beech	1	1.28	3.6	0.13	0.001	0.01
Douglas vs Be-Spr	1	0.49	1.66	0.06	0.106	1
Douglas vs Spruce	1	0.81	3.7	0.13	0.001	0.01
Be-Dgl vs Beech	1	0.81	2.26	0.07	0.03	0.3
Be-Dgl vs Be-Spr	1	0.56	1.81	0.06	0.072	0.72
Be-Dgl vs Spruce	1	1.69	6.86	0.19	0.001	0.01
Beech vs Be-Spr	1	1.49	4.57	0.13	0.001	0.01
Beech vs Spruce	1	3.24	12.27	0.29	0.001	0.01
Be-Spr vs Spruce	1	1.31	6.05	0.17	0.001	0.01

Appendix S1: Table S 9 Post hoc fitted environmental variables on the NMDS axes.

Predictor	NMDS1	NMDS2	R2	P
Vegetation volume	-0.16	-0.99	0.03	0.342
Mean gap area	-0.2	-0.98	0.18	0.001
ENL0	-0.95	-0.31	0.05	0.167
CV gap area	-0.81	-0.59	0.11	0.029
CV ICG height	-0.6	-0.8	0.13	0.009
Box-dimension	-1	-0.05	0.12	0.01

Appendix S1: Table S 10 Model summaries of linear models of functional spider responses at the plot-scale with stand type and vegetation volume as covariates. FRic = functional richness; Eve = evenness; Div = divergence.

Response	Fixed effect	Estimate	Std. Error	t-value	P
FRic	(Intercept)	0.031	0.015	2.026	0.066
FRic	stand Be-Dgl	0.03	0.019	1.554	0.146
FRic	stand Beech	-0.01	0.019	-0.54	0.599
FRic	stand Be-Spr	0.017	0.019	0.891	0.391
FRic	stand Spruce	0.025	0.019	1.329	0.208
FRic	scale(vegetation volume)	0.013	0.005	2.293	0.041
FEve	(Intercept)	0.608	0.073	8.288	<0.001

FEve	stand Be-Dgl	0.11	0.092	1.201	0.253
FEve	stand Beech	0.191	0.089	2.14	0.054
FEve	stand Be-Spr	0.015	0.09	0.164	0.872
FEve	stand Spruce	-0.084	0.089	-0.943	0.364
FEve	scale(vegetation volume)	-0.019	0.026	-0.734	0.477
FDiv	(Intercept)	0.895	0.073	12.212	<0.001
FDiv	stand Be-Dgl	-0.008	0.092	-0.082	0.936
FDiv	stand Beech	-0.077	0.089	-0.865	0.404
FDiv	stand Be-Spr	-0.009	0.09	-0.1	0.922
FDiv	stand Spruce	-0.028	0.089	-0.314	0.759
FDiv	scale(vegetation volume)	0.011	0.026	0.42	0.682

Appendix S1: Table S 11 Model summaries of linear mixed-effects models of functional spider responses at the plot-scale with environmental variables (prey abundance, mean gap area, ENL0, CV gap area, CV ICG height, Box-dimension) as fixed effects and stand type random effect. Model reduction (step function) might have reduced the number of fixed effects depending on their predictive power. FRic = functional richness; Eve = evenness; Div = divergence.

Response	Fixed effect	Estimate	Std. Error	DF	t-value	P
FRic	(Intercept)	0.043	0.008	3.511	5.421	0.008
FRic	scale(ENL0)	0.015	0.004	11.209	3.91	0.002
FRic	scale(mean gap area)	0.01	0.005	11.564	1.995	0.07
FRic	scale(CV gap area)	-0.01	0.004	9.854	-2.647	0.025
FRic	scale(CV ICG height)	0.02	0.003	9.42	7.369	<0.001
FEve	(Intercept)	0.666	0.016	14	42.903	<0.001
FEve	scale(log(prey))	-0.125	0.02	14	-6.338	<0.001
FEve	scale(CV gap area)	0.044	0.019	14	2.271	0.039
FEve	scale(CV ICG height)	-0.037	0.017	14	-2.131	0.051
FDiv	(Intercept)	0.868	0.022	17	40.179	<0.001

Appendix S1: Table S 12 Model summaries of linear models of isotopic spider responses at the plot-scale with stand type and vegetation volume as covariates. IRic = isotopic richness; Eve = evenness; Div = divergence; Uni = uniqueness.

Response	Fixed effect	Estimate	Std. Error	t-value	P
Mean $\Delta^{13}C$	(Intercept)	4.021	0.551	7.298	<0.001
Mean $\Delta^{13}C$	stand Be-Dgl	0.531	0.75	0.708	0.491
Mean $\Delta^{13}C$	stand Beech	2.025	0.721	2.81	0.015
Mean $\Delta^{13}C$	stand Be-Spr	-0.053	0.732	-0.073	0.943
Mean $\Delta^{13}C$	stand Spruce	-0.986	0.72	-1.369	0.194
Mean $\Delta^{13}C$	scale(vegetation volume)	-0.04	0.236	-0.168	0.869
Mean $\Delta^{15}N$	(Intercept)	5.687	0.682	8.339	<0.001
Mean $\Delta^{15}N$	stand Be-Dgl	0.501	0.928	0.539	0.599
Mean $\Delta^{15}N$	stand Beech	2.055	0.892	2.304	0.038
Mean $\Delta^{15}N$	stand Be-Spr	-0.321	0.905	-0.354	0.729
Mean $\Delta^{15}N$	stand Spruce	-0.404	0.892	-0.453	0.658
Mean $\Delta^{15}N$	scale(vegetation volume)	-0.1	0.292	-0.344	0.736
Minimum $\Delta^{13}C$	(Intercept)	2.953	0.65	4.54	0.001
Minimum $\Delta^{13}C$	stand Be-Dgl	0.443	0.813	0.545	0.596
Minimum $\Delta^{13}C$	stand Beech	2.42	0.794	3.05	0.01
Minimum $\Delta^{13}C$	stand Be-Spr	-0.247	0.8	-0.309	0.762
Minimum $\Delta^{13}C$	stand Spruce	-1.761	0.794	-2.219	0.047

Minimum $\Delta^{13}C$	scale(vegetation volume)	-0.213	0.23	-0.926	0.373
Minimum $\Delta^{15}N$	(Intercept)	4.324	0.837	5.169	<0.001
Minimum $\Delta^{15}N$	stand Be-Dgl	0.078	1.046	0.075	0.942
Minimum $\Delta^{15}N$	stand Beech	1.849	1.021	1.811	0.095
Minimum $\Delta^{15}N$	stand Be-Spr	-1.02	1.029	-0.992	0.341
Minimum $\Delta^{15}N$	stand Spruce	-0.696	1.021	-0.682	0.508
Minimum $\Delta^{15}N$	scale(vegetation volume)	-0.224	0.296	-0.758	0.463
Maximum $\Delta^{13}C$	(Intercept)	4.593	0.676	6.795	<0.001
Maximum $\Delta^{13}C$	stand Be-Dgl	0.878	0.845	1.039	0.319
Maximum $\Delta^{13}C$	stand Beech	2.13	0.825	2.582	0.024
Maximum $\Delta^{13}C$	stand Be-Spr	0.592	0.831	0.712	0.49
Maximum $\Delta^{13}C$	stand Spruce	-0.35	0.825	-0.424	0.679
Maximum $\Delta^{13}C$	scale(vegetation volume)	-0.17	0.239	-0.71	0.491
Maximum $\Delta^{15}N$	(Intercept)	6.361	1.079	5.894	<0.001
Maximum $\Delta^{15}N$	stand Be-Dgl	1.477	1.349	1.095	0.295
Maximum $\Delta^{15}N$	stand Beech	2.681	1.317	2.035	0.065
Maximum $\Delta^{15}N$	stand Be-Spr	1.515	1.327	1.142	0.276
Maximum $\Delta^{15}N$	stand Spruce	0.469	1.317	0.356	0.728
Maximum $\Delta^{15}N$	scale(vegetation volume)	-0.115	0.382	-0.302	0.768
Range $\Delta^{13}C$	(Intercept)	1.641	0.524	3.132	0.009
Range $\Delta^{13}C$	stand Be-Dgl	0.434	0.655	0.663	0.52
Range $\Delta^{13}C$	stand Beech	-0.29	0.639	-0.454	0.658
Range $\Delta^{13}C$	stand Be-Spr	0.839	0.644	1.303	0.217
Range $\Delta^{13}C$	stand Spruce	1.411	0.639	2.207	0.048
Range $\Delta^{13}C$	scale(vegetation volume)	0.043	0.185	0.234	0.819
Range $\Delta^{15}N$	(Intercept)	2.038	0.749	2.72	0.019
Range $\Delta^{15}N$	stand Be-Dgl	1.399	0.936	1.494	0.161
Range $\Delta^{15}N$	stand Beech	0.832	0.914	0.91	0.381
Range $\Delta^{15}N$	stand Be-Spr	2.535	0.921	2.753	0.018
Range $\Delta^{15}N$	stand Spruce	1.165	0.914	1.274	0.227
Range $\Delta^{15}N$	scale(vegetation volume)	0.109	0.265	0.411	0.688
IRic	(Intercept)	0.017	0.018	0.949	0.362
IRic	stand Be-Dgl	0.033	0.023	1.468	0.168
IRic	stand Beech	0.014	0.022	0.641	0.533
IRic	stand Be-Spr	0.024	0.022	1.062	0.309
IRic	stand Spruce	0.046	0.022	2.088	0.059
IRic	scale(vegetation volume)	0.007	0.006	1.127	0.282
IDiv	(Intercept)	0.925	0.099	9.331	<0.001
IDiv	stand Be-Dgl	-0.193	0.124	-1.561	0.145
IDiv	stand Beech	-0.156	0.121	-1.289	0.222
IDiv	stand Be-Spr	-0.226	0.122	-1.854	0.088
IDiv	stand Spruce	-0.196	0.121	-1.617	0.132
IDiv	scale(vegetation volume)	-0.008	0.035	-0.223	0.827
IEve	(Intercept)	0.687	0.19	3.612	0.004
IEve	stand Be-Dgl	-0.167	0.238	-0.703	0.495

IEve	stand Beech	-0.003	0.232	-0.014	0.989
IEve	stand Be-Spr	-0.173	0.234	-0.74	0.474
IEve	stand Spruce	-0.122	0.232	-0.528	0.607
IEve	scale(vegetation volume)	-0.003	0.067	-0.049	0.962
IUni	(Intercept)	0.659	0.16	4.134	0.001
IUni	stand Be-Dgl	-0.327	0.199	-1.642	0.127
IUni	stand Beech	0.096	0.195	0.493	0.631
IUni	stand Be-Spr	-0.065	0.196	-0.331	0.746
IUni	stand Spruce	-0.04	0.195	-0.204	0.842
IUni	scale(vegetation volume)	-0.075	0.056	-1.33	0.208

Appendix S1: Table S 13 Model summaries of linear mixed-effects models of isotopic spider responses at the plot-scale with environmental variables (prey abundance, mean gap area, ENL0, CV gap area, CV ICG height, Box-dimension) as fixed effects and stand type random effect. Model reduction (step function) might have reduced the number of fixed effects depending on their predictive power. IRic = isotopic richness; Eve = evenness; Div = divergence; Uni = uniqueness.

**Response**	**Fixed effect**	**Estimate**	**Std. Error**	**DF**	**t-value**	**P**
Mean $\Delta^{13}C$	(Intercept)	4.341	0.181	17	23.931	<0.001
Mean $\Delta^{13}C$	scale(log(prey))	-1.066	0.186	17	-5.723	<0.001
Mean $\Delta^{15}N$	(Intercept)	6.072	0.252	17	24.056	<0.001
Mean $\Delta^{15}N$	scale(log(prey))	-0.86	0.259	17	-3.316	0.004
Minimum $\Delta^{13}C$	(Intercept)	3.222	0.233	16	13.837	<0.001
Minimum $\Delta^{13}C$	scale(log(prey))	-1.362	0.239	16	-5.702	<0.001
Minimum $\Delta^{15}N$	(Intercept)	4.43	0.297	2.951	14.939	0.001
Minimum $\Delta^{15}N$	scale(log(prey))	-0.892	0.298	5.749	-2.998	0.025
Maximum $\Delta^{13}C$	(Intercept)	5.375	0.16	16	33.686	<0.001
Maximum $\Delta^{13}C$	scale(log(prey))	-1.026	0.164	16	-6.266	<0.001
Maximum $\Delta^{15}N$	(Intercept)	7.77	0.337	16	23.086	<0.001
Maximum $\Delta^{15}N$	scale(log(prey))	-0.752	0.345	16	-2.177	0.045
Range $\Delta^{13}C$	(Intercept)	2.052	0.396	3.459	5.175	0.01
Range $\Delta^{13}C$	scale(mean gap area)	0.456	0.188	11.768	2.428	0.032
Range $\Delta^{13}C$	scale(CV gap area)	-0.503	0.169	10.35	-2.965	0.014
Range $\Delta^{13}C$	scale(CV ICG height)	0.453	0.117	9.831	3.881	0.003
Range $\Delta^{15}N$	(Intercept)	3.319	0.33	3.631	10.052	0.001
Range $\Delta^{15}N$	scale(box-dimension)	0.495	0.234	14.553	2.119	0.052
Range $\Delta^{15}N$	scale(CV gap area)	-0.519	0.233	14.498	-2.225	0.042
IRic	(Intercept)	0.042	0.006	2.166	7.094	0.016
IRic	scale(CV ICG height)	0.014	0.006	14.194	2.462	0.027
IDiv	(Intercept)	0.755	0.026	15	29.053	<0.001
IDiv	scale(box-dimension)	-0.068	0.027	15	-2.561	0.022
IDiv	scale(mean gap area)	0.061	0.026	15	2.302	0.036
IEve	(Intercept)	0.584	0.056	17	10.426	<0.001
IUni	(Intercept)	0.589	0.065	4.255	9.014	0.001

References

Arachnologische Gesellschaft e. V. 2021: Wiki des Spinnen-Forums. arages.de.

Arseniou, G., D. W. MacFarlane, and D. Seidel. 2021. Measuring the Contribution of Leaves to the Structural Complexity of Urban Tree Crowns with Terrestrial Laser Scanning. Remote Sensing 13:2773.

Brokaw, N. V. L. 1982. The Definition of Treefall Gap and Its Effect on Measures of Forest Dynamics. Biotropica 14:158.

Ehbrecht, M., P. Schall, J. Juchheim, C. Ammer, and D. Seidel. 2016. Effective number of layers: A new measure for quantifying three-dimensional stand structure based on sampling with terrestrial LiDAR. Forest Ecology and Management 380:212–223.

Fink, E. L. 2009. The FAQs on Data Transformation. Communication Monographs 76:379–397.

Fox, J., and S. Weisberg. 2018. An R companion to applied regression. Sage publications.

Fry, B. 2006. Stable isotope ecology. Springer, New York.

Kuznetsova A, Brockhoff PB, Christensen RHB. 2017. "lmerTest Package: Tests in Linear Mixed Effects Models." Journal of Statistical Software, 82(13), 1–26. doi:10.18637/jss.v082.i13.

Langel, R., and J. Dyckmans. 2014. Combined ^{13}C and ^{15}N isotope analysis on small samples using a near-conventional elemental analyzer/isotope ratio mass spectrometer setup: Combined ^{13}C and ^{15}N isotope analysis on small samples via µEA/IRMS. Rapid Communications in Mass Spectrometry 28:1019–1022.

Layman, C. A., D. A. Arrington, C. G. Montaña, and D. M. Post. 2007. Can stable isotope ratios provide for community-wide measures of trophic structure? Ecology 88:42–48.

Perkins, M. J., R. A. McDonald, F. J. F. van Veen, S. D. Kelly, G. Rees, and S. Bearhop. 2013. Important impacts of tissue selection and lipid extraction on ecological parameters derived from stable isotope ratios. Methods in Ecology and Evolution:n/a-n/a.

Roussel, J.-R., D. Auty, N. C. Coops, P. Tompalski, T. R. H. Goodbody, A. S. Meador, J.-F. Bourdon, F. De Boissieu, and A. Achim. 2020. lidR: An R package for analysis of Airborne Laser Scanning (ALS) data. Remote Sensing of Environment 251:112061.

Seidel, D. 2018. A holistic approach to determine tree structural complexity based on laser scanning data and fractal analysis. Ecology and Evolution 8:128–134.

Silva, C. A., R. Valbuena, E. R. Pinagé, M. Mohan, D. R. A. Almeida, E. North Broadbent, W. S. W. M. Jaafar, D. Papa, A. Cardil, and C. Klauberg. 2019. F OREST G AP R: An R Package for forest gap analysis from canopy height models. Methods in Ecology and Evolution 10:1347–1356.

World Spider Catalog. 2022. World Spider Catalog, Verion 23.0. https://doi.org/10.24436/2
